# Tuina Alleviates Anxiety‐Like Behaviors Associated With Neuropathic Pain Through the Modulation of Synaptic Plasticity in the Anterior Cingulate Cortex

**DOI:** 10.1002/brb3.70965

**Published:** 2025-10-15

**Authors:** Rentuya Na, Yue Xu, Tianyuan Yu, Yufeng Gao, Yingqi Zhang, Jiawang Yan, Hongzheng Zhang

**Affiliations:** ^1^ School of Acupuncture‐Moxibustion and Tuina Beijing University of Chinese Medicine Beijing China; ^2^ Department of Neurology Affiliated Hospital of Inner Mongolia Minzu University Tongliao China

**Keywords:** anterior cingulate cortex, anxiety‐like behaviors, neuropathic pain, synaptic plasticity, Tuina

## Abstract

**Background:**

As the disease progresses, neuropathic pain (NP) is often accompanied by anxiety‐like behaviors, which are associated with plastic changes in the synaptic structure and function within the anterior cingulate cortex (ACC). Tuina is a traditional Chinese manual therapy. This study aims to investigate the effects of Tuina on pain behaviors and related anxiety‐like behaviors in rats with the chronic constriction injury (CCI) model, as well as its impact on synaptic plasticity in the ACC.

**Methods:**

The rats were randomly divided into three groups: Sham, CCI, and Tuina. An NP comorbid anxiety model was established by inducing CCI in the right sciatic nerve. The pain‐related behaviors and anxiety‐like behaviors in rats were assessed using the mechanical withdrawal threshold (MWT), thermal withdrawal latency (TWL), open field test (OFT), and elevated plus maze (EPM). Supplementary Material The changes in the synaptic ultrastructure within the ACC were examined using transmission electron microscopy (TEM). The changes in excitatory synaptic transmission (synaptic function) within the ACC were investigated using the whole‐cell patch‐clamp technique. The expression levels of the postsynaptic proteins PSD‐95, GluN2B, GluR1, and CaMKII in the ACC were analyzed using Western blot.

**Results:**

Tuina significantly elevated the pain threshold in CCI rats and mitigated their anxiety‐like behaviors. Tuina alleviated the abnormal remodeling of synaptic structures in the ACC and mitigated the CCI‐mediated enhancement of excitatory synaptic transmission. Tuina may exert its analgesic and anxiolytic effects by modulating the expression of postsynaptic proteins PSD‐95, glutamate receptor GluN2B, GluR1, and the downstream protein CaMKII in the ACC.

**Conclusion:**

Tuina effectively alleviates pain sensitivity and anxiety‐like behaviors in CCI model rats while modulating abnormal synaptic remodeling in the ACC. This study provides fundamental experimental evidence supporting Tuina as a potential therapeutic option for NP associated with anxiety‐like behaviors.

## Graphical abstract

Tuina alleviates CCI‐induced neuropathic pain and anxiety‐like behaviors, and counteracts the enhancement of excitatory synaptic transmission and abnormal synaptic plasticity, potentially by modulating GluN2B/PSD‐95/CaMKII/GluR1 signaling.

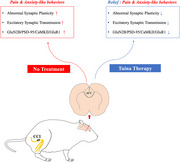

## 1 Introduction

Neuropathic pain (NP) is a pain condition resulting from lesions or diseases affecting the somatosensory nervous system. Its characteristics include hyperalgesia and allodynia, among others (Jensen et al. 2011). NP is often associated with negative emotions, which can in turn exacerbate the perception of pain (Velly and Mohit 2018; Vlaeyen and Linton 2000). Clinical studies have demonstrated that with the prolonged duration of pain, approximately 20.3% of patients with chronic peripheral NP develop anxiety‐like behaviors (Radat et al. 2013). Current pharmacological treatments exhibit limited efficacy for pain relief and antianxiety effects, while also inducing a range of adverse reactions. Thus, there is a critical need to develop safer and more effective therapeutic approaches.

Tuina therapy, a traditional physical treatment method rooted in traditional Chinese medicine, has been practiced in China for thousands of years. It involves the application of various techniques, such as pressing, rubbing, and tapping, to the body surface with the aim of regulating physiological and pathological states, thereby facilitating disease recovery (M. Y. Wang et al. 2008). Tuina is widely applied for pain relief owing to its safety, effectiveness, and minimal side effects (P. Ma et al. 2025; Nie et al. 2019; Q. Ma et al. 2024). In recent years, Tuina has been demonstrated to effectively alleviate anxiety associated with nonspecific chronic neck pain, insomnia, and even allergic airway inflammation (Cheng et al. 2022; Z. Wang et al. 2024; Liu et al. 2022). However, it remains unclear whether Tuina can serve as a preventive or therapeutic approach for anxiety‐like behaviors induced by NP.

It is widely recognized that the anterior cingulate cortex (ACC) plays a critical role in pain perception and emotional regulation (Vogt 2005). Studies have demonstrated that NP induces abnormal activation of pyramidal neurons in the ACC region, significantly enhances synaptic transmission, and causes abnormal plasticity in synaptic structures, thereby exacerbating pain perception and intensifying anxiety‐related responses (T. Z. Wang et al. 2023; Y. D. Li et al. 2024; Xu et al. 2008). Nonspecific silencing of ACC neurons or inhibition of abnormal synaptic plasticity can reduce hyperalgesic behaviors and anxiety‐like behaviors (Wen et al. 2022; X. H. Li et al. 2021). These findings indicate that modulating excitatory synaptic transmission and structural abnormal plasticity in the ACC is a critical approach for managing the comorbidity of NP and anxiety. Therefore, we examined the effects of Tuina on excitatory synaptic transmission and the ultrastructural characteristics of ACC neurons.

The overactivation of glutamate receptors in ACC neurons represents a critical mechanism contributing to the augmentation of excitatory synaptic transmission (Xu et al. 2008). GluN2B serves as a regulatory subunit of the glutamate NMDA receptor, playing a critical role in modulating pain perception and mediating synaptic responses (Wei et al. 2001). GluR1 serves as a regulatory subunit of the glutamate AMPA receptor and plays a key role in mediating rapid excitatory synaptic transmission within the central nervous system (Lee et al. 2003). Studies have demonstrated that the overactivation of GluN2B induces calcium ion influx, which triggers CaMKII phosphorylation and initiates a cascade reaction, thereby modulating the activity of AMPA glutamate receptors. This process not only mediates the enhancement of excitatory postsynaptic currents (EPSCs) but also contributes to ACC synaptic plasticity, ultimately amplifying pain transmission and influencing emotional responses (Xu et al. 2008; Zhuo 2009; Hu et al. 2006). This suggests that the GluN2B/CaMKII/GluR1 signaling pathway plays a critical role as a therapeutic target for modulating synaptic plasticity in the ACC brain region, thereby alleviating NP and associated anxiety‐like behaviors.

In this study, we evaluated the analgesic and anxiolytic effects of Tuina using mechanical withdrawal threshold (MWT), thermal withdrawal latency (TWL), open field test (OFT), and elevated plus maze (EPM) as assessment tools. We further employed transmission electron microscopy (TEM) and whole‐cell patch‐clamp techniques to investigate the effects of Tuina on excitatory synaptic transmission and the aberrant remodeling of synaptic structures. Finally, Western blot was utilized to evaluate the impact of Tuina on the expression levels of synaptic‐related receptors and molecules, including GluN2B, CaMKII, GluR1, and PSD‐95.

## 2 Materials and Methods

### 2.1 Animals

A total of 36 male Sprague–Dawley rats, weighing 200 ± 10 g, were procured from SPF (Beijing) Biotechnology Co. Ltd. (Experimental Animal Production License No.: SCXK [Jing] 2024‐0001). The rats were raised at the Animal Experiment Center of Beijing University of Chinese Medicine (5 rats per cage). Environmental conditions included a temperature of 25 ± 2°C and a strict 12‐h light‐dark cycle. The rats were randomly divided into three groups (*n* = 12 for each group): Sham, CCI, and Tuina. Prior to the start of the experiment, they underwent a 1‐week acclimatization period. All experimental procedures were approved by the Ethics Committee of Beijing University of Chinese Medicine (License No.: BUCM‐2024090203‐3127) and adhered strictly to the National Institutes of Health guide for the care and use of laboratory animals. The number of rats was minimized, and efforts were made to reduce their suffering.

### 2.2 Modeling Surgery

**FIGURE 1:**
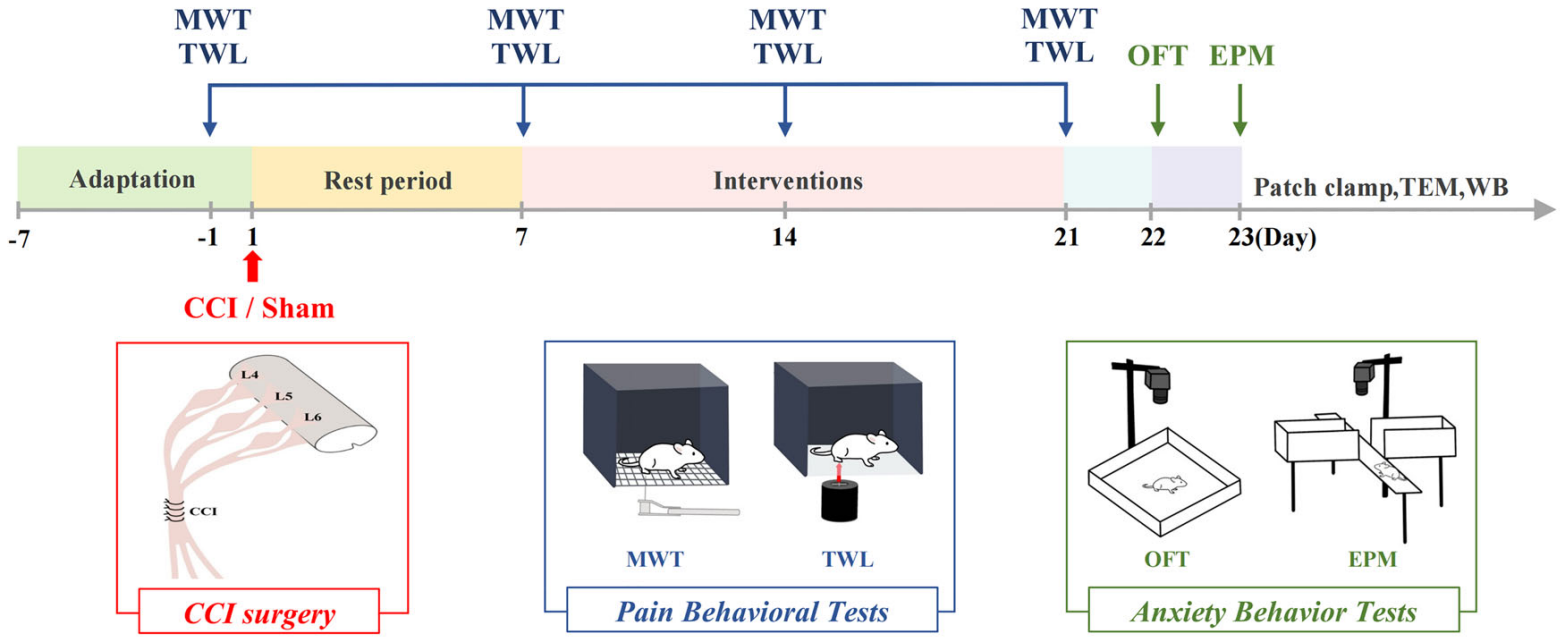
Schematic of the research protocol followed in this study.

The CCI and Tuina groups underwent CCI modeling surgery, as previously described (Bennett and Xie 1988) (Figure 1).

The animals were fasted for 12 h prior to surgery. The rats were fixed in the prone position, and gas anesthesia was performed with isoflurane. The right sciatic nerve was exposed. Four 4–0 loose chromic gut ligatures were loosely placed at the proximal end of the sciatic nerve bifurcation prior to wound closure. The Sham group underwent the same surgical procedure as the CCI group, but only the sciatic nerve was exposed without ligation. The patients were fasted for 12 h after surgery, and the wound condition and foot changes were observed regularly during the experiment.

### 2.3 Intervention Methods

All interventions started on Day 7 after CCI modeling and lasted for 14 days.

Tuina group: The Tuina manipulation instrument (Chinese invention patent number: ZL202320511277.5) was employed to stimulate the ipsilateral Yingmen (BL37), Yanglingquan (GB34), and Chengshan (BL57) acupoints in rats using point‐pressing, plucking, and kneading manipulation (Zhang et al. 2024). Parameters were set as follows: stimulation intensity at 4N, operation frequency at 60 times per minute, each acupoint and manipulation stimulated for 1 min, with a total treatment time of 9 min per day.

Sham group and CCI group: Grasping restraint for 9 min.

### 2.4 Behavior Test

The MWT was used to assess recovery of mechanical pain sensation. The procedure was as follows: Rats were placed in a cage with a metal mesh floor 20 min before testing and tested once calm. The probe of an electronic pain meter was positioned at the center of the right hind paw's plantar surface. Pressure was gradually increased until the rat withdrew its paw or licked it, and the value was recorded. This was repeated three times with 10‐min intervals.

The TWL was used to evaluate recovery of thermal pain sensation (Figure 1). The procedure was as follows: Rats were placed in a glass‐bottomed cage 20 min before testing and tested once calm. The infrared source was directed to the center of the right hind paw. Stimulation was initiated, and the value was recorded upon withdrawal or licking. This was repeated three times with 10‐min intervals. The MWT, TWL, and Tuina interventions were all performed with at least 2‐h intervals between each procedure.

The EPM test was used to assess anxiety‐like behaviors. Animals were acclimated in a quiet, dimly lit room 60 min before testing. The maze was 60 cm high and consisted of two open arms (10 × 50 cm), two closed arms (10 × 50 cm), and a central area (10 × 10 cm). Rats were placed in the center facing an open arm, and their movement was recorded for 5 min. Time spent in the open arms and total distance traveled were analyzed. Less time in the open arms indicated higher anxiety. After each trial, the maze was cleaned with 75% ethanol to remove excrement and odors, minimizing olfactory interference.

The OFT was used to assess anxiety‐like behaviors. The testing room was dimly lit and kept quiet during the experiment. The floor of the 100 × 100 × 50 cm black open field box was divided into 16 equal grids: 4 central grids formed the center zone, and the remaining 12 constituted the peripheral zone. Rats were placed in the center and recorded for 10 min. Time spent in the center and total distance traveled were analyzed; less time and shorter distance indicated higher anxiety.

### 2.5 Tissue Selection

Following the 14‐day intervention and final behavioral EPM tests (24 ± 1 h post‐testing), rats (*n* = 12/group) were anesthetized and humanely euthanized. Brains were immediately dissected, with the ACC processed sequentially for: (1) acute slice preparation for whole‐cell patch‐clamp recordings (*n* = 3), (2) TEM analysis using 1–2 mm fragments fixed in TEM fixative solution and stored at 4°C (*n* = 3), (3) Western blotting with snap‐frozen samples stored at −80°C (*n* = 3), and (4) whole‐brain perfusion fixation with 4% paraformaldehyde for archival preservation (*n* = 3). All procedures were completed within 30 min post‐euthanasia to ensure optimal tissue preservation.

### 2.6 Whole‐Cell Patch Clamp

The whole‐cell patch‐clamp experiments were performed as previously described (X. Wu, Gao et al. 2023).

#### 2.6.1 Preparation of Acute Brain Slices

The rats were anesthetized with isoflurane and their brains were quickly extracted. The brain was placed in a cold slicing solution with low sodium and high sucrose. Coronal brain slices of 300 µm thickness were prepared using a vibratome (VT1200S). Incubation: Following preparation, the brain slices were transferred to an incubation chamber containing artificial cerebrospinal fluid (ACSF) and incubated at 37°C for 1 h. Subsequently, they were maintained at room temperature until electrophysiological recording was completed. The ACSF composition (in mM) was as follows: 125 NaCl, 2.5 KCl, 2 CaCl_2_, 1 MgCl_2_, 26 NaHCO_3_, 1.25 NaH_2_PO_4_, and 25 Glucose. The solution was continuously bubbled with a mixture of 95% O_2_ and 5% CO_2_ to ensure full oxygenation and pH stabilization.

#### 2.6.2 Brain Slice Fixation

The brain slices were placed in the recording chamber and continuously perfused with ACSF at a flow rate of 2–3 mL/min, maintaining a temperature of 32°C–34°C throughout the procedure.

#### 2.6.3 Patch‐Clamp Recording

Glass microelectrodes were used to establish contact with the cell membrane, forming a high‐resistance (GΩ‐level) seal. Enter the whole‐cell recording mode to record miniature EPSCs (mEPSCs). TTX (1 µM), bicuculline (10 µM), and CGP 54626 hydrochloride (2 µM) were added to the extracellular solution to block action potentials and GABAA receptor‐mediated responses. A cesium‐based intracellular solution was used, with the clamp voltage maintained at −60 mV. The cesium‐based intracellular solution contained the following (in mM): 135 CsMeSO_3_, 10 CsCl, 10 HEPES, 0.2 EGTA, 4 MgATP, and 0.3 Na_2_GTP. The pH was adjusted to 7.2–7.3.

#### 2.6.4 Data Acquisition

Current signals were recorded using a data acquisition system, and mEPSCs were detected and analyzed using Clampfit software.

### 2.7 Synaptic Ultrastructure Analysis by TEM

The TEM experiments were conducted following a previously established protocol (Yang et al. 2023; Jia et al. 2022). Briefly, fresh ACC tissue was dissected into 1–2 mm fragments and fixed in TEM fixative, followed by storage at 4°C. The samples were then dehydrated, embedded, and sectioned to approximately 80 nm using an ultrathin microtome. Sections were dried overnight at room temperature. Observations and imaging were performed using a transmission electron microscope. Quantitative analysis of synaptic cleft width, presynaptic active zone length, and postsynaptic dense substance thickness was performed using ImageJ software.

### 2.8 Western Blot

The ACC tissues were lysed on ice, and the supernatant was collected via centrifugation. Protein concentration was determined using the BCA protein quantification kit. Subsequently, gel electrophoresis, membrane transfer, and blocking were carried out. The membranes were incubated overnight at 4°C with primary antibodies against PSD‐95 (Affinity, China), GluN2B (Servicebio, China), p‐CaMKII (Affinity, China), and GluR1 (Affinity, China). Following three washes of the membrane, the secondary antibody was added, and the membranes were incubated at room temperature for 2 h. Immunoreactive bands were visualized using the ECL chemiluminescent solution, and the gray values were quantified using ImageJ software.

### 2.9 Statistical Analysis

All statistical analyses were performed using GraphPad Prism 9.5.1, and data are presented as mean ± standard error of the mean (SEM). After confirming that the data conformed to a normal distribution via the Shapiro–Wilk test, appropriate statistical tests were selected: For repeated‐measures data (e.g., MWT, TWL), two‐way repeated‐measures ANOVA was applied (Mauchly's test of sphericity was conducted, and Greenhouse–Geisser correction was used when sphericity was violated); For one‐way data, one‐way ANOVA was used when variances were homogeneous, and Welch's ANOVA was employed when variances were heterogeneous. When ANOVA results were significant, Tukey's post hoc test was used for homogeneous variances, and Dunnett's T3 test was applied for heterogeneous variances. The significance level was set at *p* < 0.05.

## 3 Results

### 3.1 Tuina Treatment Effectively Alleviated Pain Hypersensitivity Induced by CCI

To assess the analgesic efficacy of Tuina, MWT and TWL tests were performed on Day 0 (7 days post‐CCI), Day 7 (14 days post‐CCI), and Day 14 (21 days post‐CCI) of the Tuina intervention.

**FIGURE 2:**
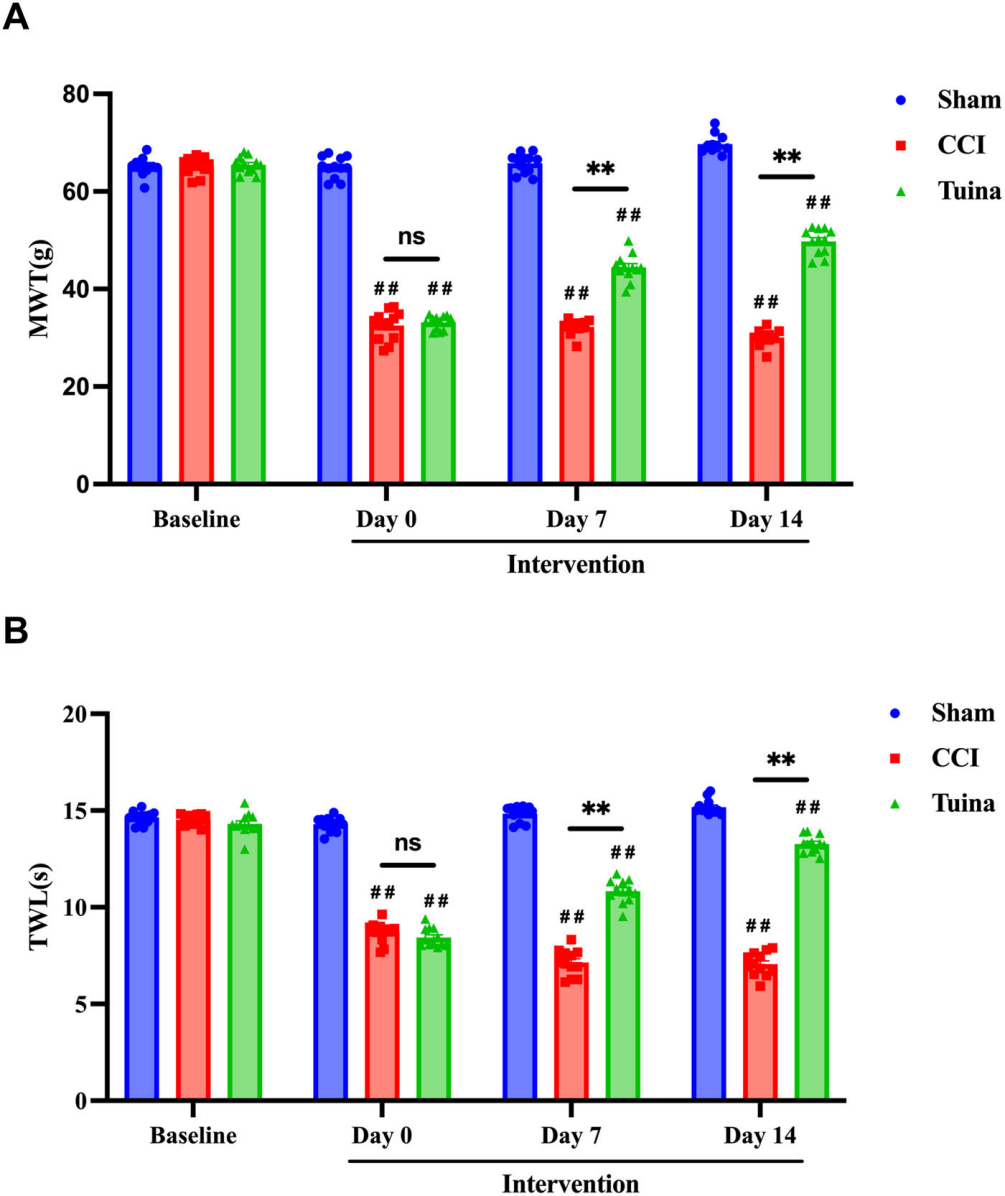
Tuina treatment effectively alleviated pain hypersensitivity induced by CCI (*n* = 12). (A) The MWT results: *F*(2, 33) = 2024, *p* < 0.001. (B) The TWL results: *F*(2, 33) = 1196, *p* < 0.001. Data were analyzed by two‐way repeated‐measures ANOVA. ^##^ *p* < 0.01 versus Sham. ^**^ *p* < 0.01 versus CCI.

Figure 2A,B shows that baseline MWT and TWL values did not differ significantly across experimental groups. Compared with the Sham group, both the CCI and Tuina groups exhibited significantly reduced MWT and TWL on intervention Day 0 (*p* < 0.01), indicating pronounced mechanical and thermal hyperalgesia. As the intervention progressed, the Tuina group exhibited significantly higher MWT and TWL levels compared to the CCI group on both Days 7 and 14 of the intervention (*p* < 0.01). The results show that Tuina intervention significantly enhances the pain threshold in CCI rats and effectively alleviates their hyperalgesic responses.

### 3.2 Tuina Significantly Reduces Anxiety‐Like Behaviors Induced by CCI

Given that chronic pain not only induces pain hypersensitivity but may also provoke anxiety, the anxiety‐like behaviors of the rats were evaluated using OFT and EPM tests on Days 22 and 23 post‐CCI surgery.

**FIGURE 3:**
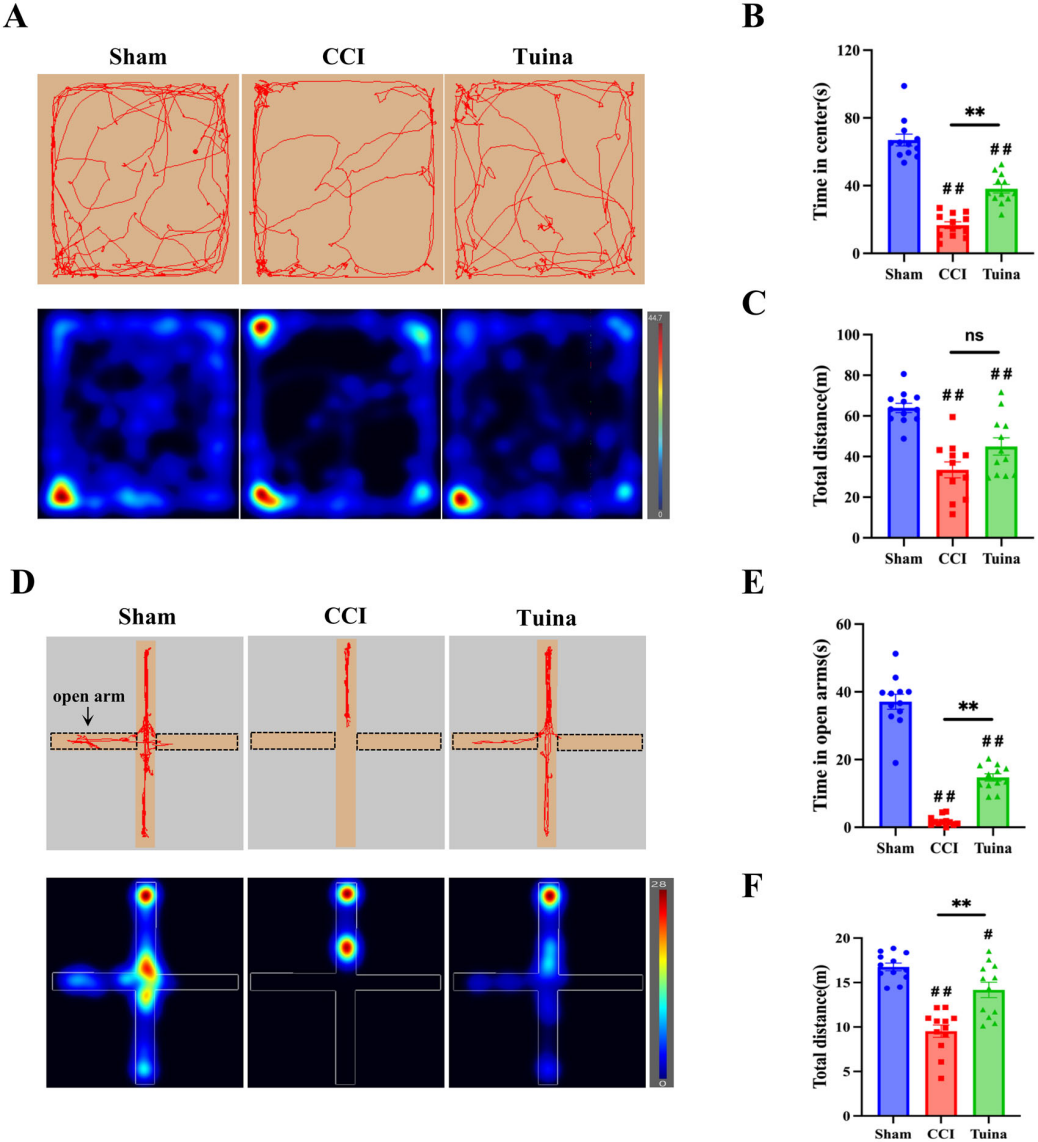
Tuina significantly reduces anxiety‐like behaviors induced by CCI (*n* = 12). (A) Representative movement trajectories and heatmaps in the OFT. (B) OFT time in center: one‐way ANOVA, *F*(2, 33) = 84.28, *p* < 0.001. (C) OFT total distance: one‐way ANOVA, *F*(2, 33) = 18.50, *p* < 0.001. (D) Representative movement trajectories and heatmaps in the EPM. (E) EPM time in open arms: Welch's ANOVA, W (2.000, 16.98) = 166.2, *p* < 0.0001. (F) EPM total distance: one‐way ANOVA, *F*(2, 33) = 28.41, *p* < 0.001. ^#^ *p* < 0.05 versus Sham. ^##^ *p* < 0.01 versus Sham. ^**^ *p* < 0.01 versus CCI.

OFT results (Figure 3A,C) show that, compared with the Sham group, both the total distance traveled and the time spent in the central area were significantly decreased in CCI rats (*p* < 0.01). Compared with the CCI group, the time spent in the central zone was significantly increased in the Tuina group (*p* < 0.01), whereas the total distance traveled exhibited a nonsignificant increasing trend (*p* > 0.05).

EPM results (Figure 3D,F) show that, compared with the Sham group, the time spent in the open arms of rats in the CCI group was significantly shortened (*p* < 0.01); while compared with the CCI group, the time spent in the open arms of rats in the Tuina group was significantly prolonged (*p* < 0.01). Meanwhile, compared with the Sham group, the total movement distance of rats in the CCI group was significantly reduced (*p* < 0.01); compared with the CCI group, the total movement distance of rats in the Tuina group was significantly increased (*p* < 0.01).

### 3.3 Tuina Can Ameliorate CCI‐Mediated Abnormal Synaptic Plasticity in the ACC

**FIGURE 4:**
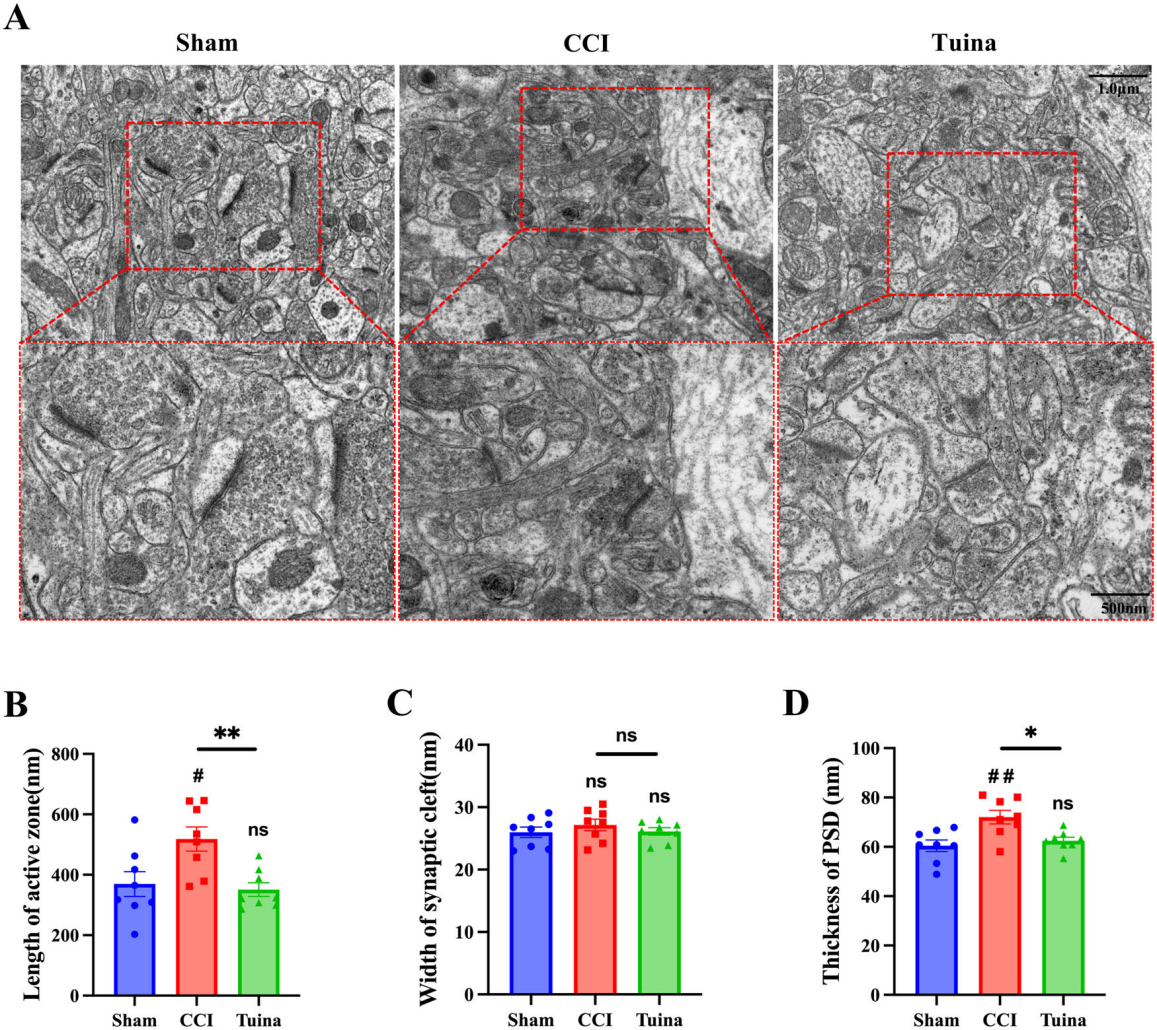
The effect of Tuina on the synaptic ultrastructural morphology in the ACC of CCI rats (*n* = 8 slices from 3 rats). (A) Representative TEM images showing synaptic ultrastructure in the ACC. (B) Length of active zone: *F*(2, 21) = 6.644, *p* = 0.006. (C) Width of synaptic cleft: *F*(2, 21) = 0.6610, *p* = 0.53. (D) Thickness of the postsynaptic densities (PSD): *F*(2, 21) = 7.660, *p* = 0.003. Data were analyzed by one‐way ANOVA. ^#^ *p* < 0.05 versus Sham. ^##^ *p* < 0.01 versus Sham. ^*^ *p* < 0.05 versus CCI. ^**^ *p* < 0.01 versus CCI.

As shown in Figure 4, compared with the Sham group, the CCI group exhibited a greater number of abnormal synapses, characterized by increased synaptic active zone length, blurred synaptic interface curvature, and elevated thickness of the postsynaptic density. These morphological rearrangements may contribute to enhanced abnormal signal transmission between neurons. Compared with the CCI group, the synaptic ultrastructure in the Tuina group was improved, as evidenced by clearer presynaptic and postsynaptic membranes, a reduced synaptic active zone length, and decreased thickness of the postsynaptic density. The results demonstrate that Tuina can ameliorate CCI‐induced abnormal synaptic remodeling in the ACC.

### 3.4 Tuina Attenuated the CCI‐Mediated Enhancement of Excitatory Synaptic Transmission in ACC Neurons

**FIGURE 5:**
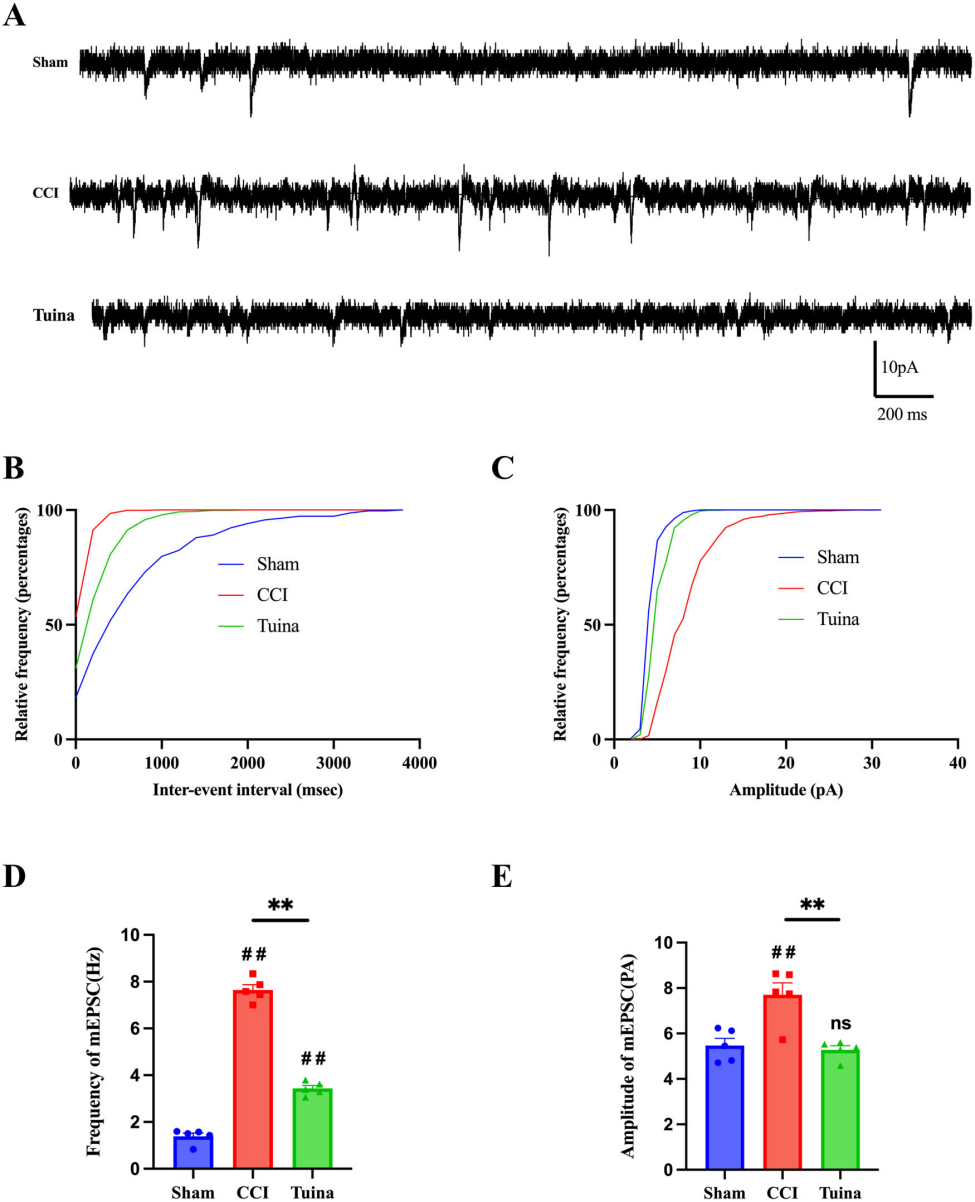
The effect of Tuina on excitatory synaptic transmission within the ACC of CCI rats (*n* = 5 cells from 3 rats). (A) The sample traces of the ACC neurons in each group. (B,C) Cumulative distribution of the frequency and amplitude of mEPSCs. (D) Frequency of mEPSC: *F*(2, 12) = 355.2, *p* < 0.001. (E) Amplitude of mEPSC: *F*(2, 12) = 13.30, *p* < 0.001. Data were analyzed by one‐way ANOVA. ^##^ *p* < 0.01 versus Sham. ^**^ *p* < 0.01 versus CCI.

As shown in Figure 5, compared with the Sham group, both the amplitude and frequency of mEPSCs in ACC neurons were significantly increased in the CCI group (*p* < 0.01). Following Tuina intervention, both the amplitude and frequency of mEPSCs were significantly decreased (*p* < 0.01). The results demonstrate that Tuina intervention can attenuate CCI‐induced enhancement of excitatory synaptic transmission in the ACC.

### 3.5 The GluN2B/CaMKII/GluR1 Pathway Is a Key Mechanism Underlying Tuina's Analgesic and Antianxiety Effects

**FIGURE 6:**
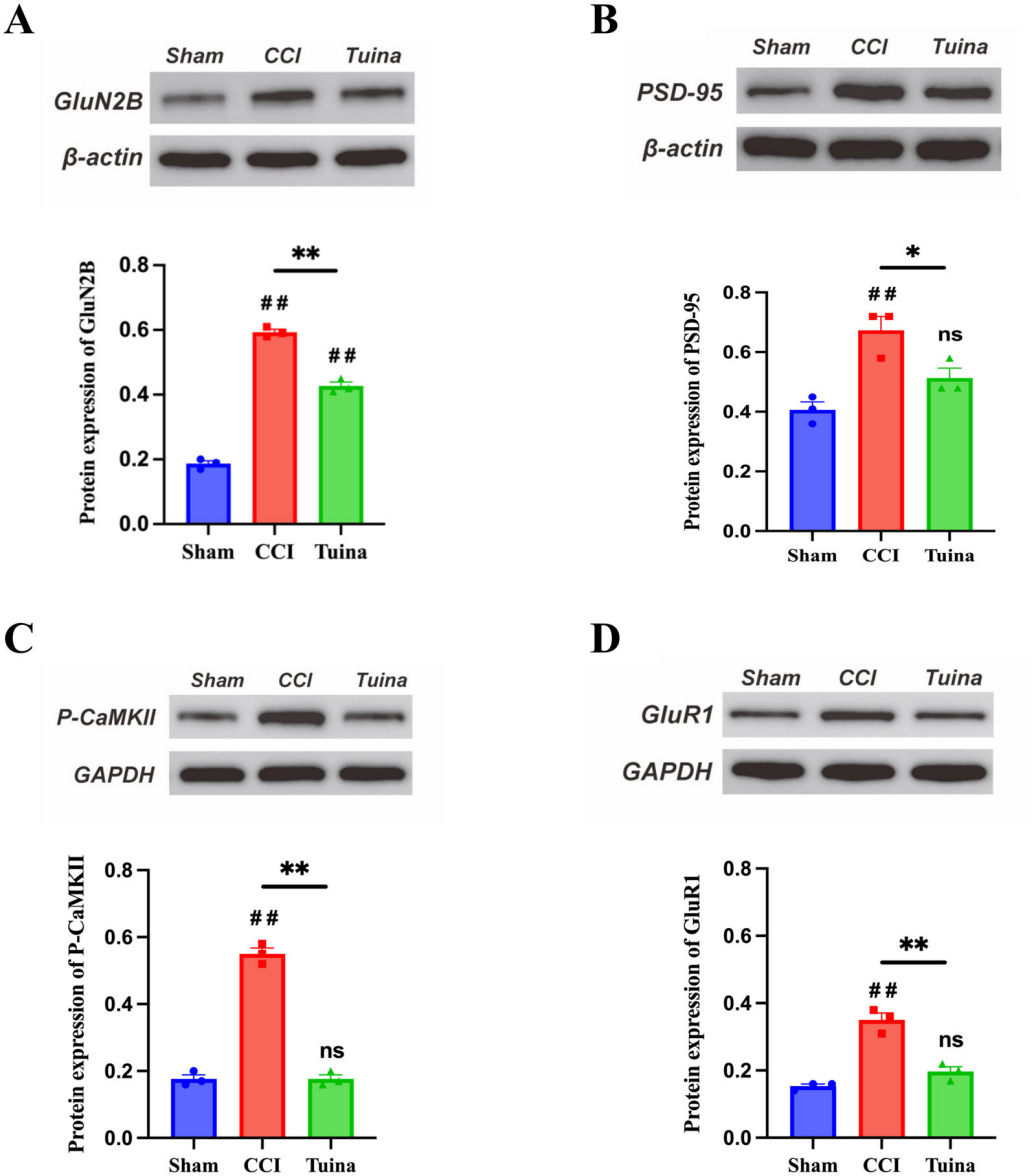
The effect of Tuina on the expression levels of synaptic‐related receptors and signaling molecules in the ACC of CCI rats (*n* = 3 biological replicates). (A) Representative images and analysis of Western blots of GluN2B: *F*(2, 6) = 417.9, *p* < 0.001. (B) Representative images and analysis of Western blots of PSD‐95: *F*(2, 6) = 13.62, *p* = 0.006. (C) Representative images and analysis of Western blots of p‐CaMKII: *F*(2, 6) = 236.7, *p* < 0.001. (D) Representative images and analysis of Western blots of GluR1: *F*(2, 6) = 46.50, *p* < 0.001. Data were analyzed by one‐way ANOVA. ^##^ *p* < 0.01 versus Sham. ^*^ *p* < 0.05 versus CCI. ^**^ *p* < 0.01 versus CCI.

As shown in Figure 6, the protein levels of PSD‐95, GluN2B, p‐CaMKII, and GluR1 were significantly higher in the CCI group than in the Sham group (*p* < 0.01). Compared with the CCI group, the protein levels of PSD‐95, GluN2B, p‐CaMKII, and GluR1 were significantly reduced in the Tuina group (*p* < 0.05 or *p* < 0.01). These findings indicate that Tuina's analgesic and antianxiety effects may involve the suppression of abnormal activation of postsynaptic glutamate receptors in the ACC and their downstream signaling pathways.

## 4 Discussion

Anxiety represents one of the most prevalent comorbid complications associated with NP (Velly and Mohit 2018). The CCI model, characterized by its reliability and stability, is a well‐established experimental paradigm that has been extensively utilized in studies investigating the underlying mechanisms of chronic pain and anxiety comorbidity (Bennett and Xie 1988). Studies have shown that CCI‐induced anxiety‐like behaviors typically manifest 2–4 weeks following surgical intervention (Naeimi Ghahroodi et al. 2025; Fonseca‐Rodrigues et al. 2021). The OFT and EPM are widely employed behavioral paradigms for assessing anxiety‐like behaviors in rodents. Both tests capitalize on the animals’ innate aversion to open and unfamiliar environments, wherein exploratory behavior in aversive zones (e.g., the center in OFT and open arms in EPM) serves as a valid indicator of anxiety‐related states (Prut and Belzung 2003; Walf and Frye 2007). Our behavioral findings demonstrate that rats subjected to CCI exhibited significant hyperalgesia and pronounced anxiety‐like behaviors. Following Tuina intervention, a marked increase was observed in both the MWT and TWL. Furthermore, Tuina‐treated rats spent significantly more time in the central area of the OFT and the open arms of the EPM, indicating that Tuina administration not only alleviated NP but also mitigated comorbid anxiety‐like behaviors. It is noteworthy, however, that no significant difference was detected in the total distance traveled during the OFT between the Tuina and CCI groups. We interpret this result to suggest that total locomotion represents a composite measure influenced by both emotional state and motor capacity. Consequently, future investigations should incorporate more specific motor function assessments—such as gait analysis or treadmill endurance tests—to better dissociate the specific effects of Tuina on emotional regulation versus those on functional motor recovery.

Alterations in synaptic transmission efficacy and ultrastructural features of ACC neurons serve as key drivers in the induction of persistent pain hypersensitivity and associated emotional responses (T. Z. Wang et al. 2023; Y. D. Li et al. 2024; Xu et al. 2008; Zhuo 2013). Studies have shown that nerve injury enhances excitatory synaptic transmission in the ACC, which in turn amplifies the responsiveness of ACC neurons to subsequent sensory stimuli (X. Y. Li et al. 2010; Toyoda et al. 2009). Spontaneous mEPSCs, recorded in the presence of presynaptic action potential blockade, serve as critical indicators for assessing synaptic transmission. Our results demonstrate that, compared to the Sham group, both the frequency and amplitude of mEPSCs in layer II/III neurons of the ACC were significantly elevated in the CCI group, consistent with previous reports (Toyoda et al. 2009; L. J. Wu and Zhuo 2009). Notably, compared to the CCI group, both the frequency and amplitude of mEPSCs were significantly reduced following Tuina intervention, indicating that Tuina attenuated the CCI‐induced enhancement of excitatory synaptic transmission in the ACC. In addition, TEM was employed to investigate alterations in synaptic ultrastructure within the ACC. Our findings revealed that, compared to the CCI group, Tuina intervention ameliorated the CCI‐induced rearrangement of synaptic ultrastructure. Our findings suggest that Tuina may exert its analgesic and anxiolytic effects through the modulation of synaptic transmission efficacy and neuronal ultrastructural alterations in CCI rats.

During NP, the expression levels of the glutamatergic receptor NMDA subunit GluN2B and the AMPA subunit GluR1 in the ACC are significantly upregulated. Moreover, synaptic transmission currents mediated by GluN2B receptors are increased. These changes enhance synaptic transmission and contribute to synaptic plasticity (Xu et al. 2008; L. J. Wu and Zhuo 2009). CaMKII, as a downstream effector of GluN2B, constitutes a key protein component of the postsynaptic density. It is capable of detecting changes in postsynaptic calcium ion concentrations and maintaining its enzymatic activity through autophosphorylation. This molecular mechanism underlies its critical role in modulating glutamatergic synaptic plasticity (Zhu et al. 2020; Zhou et al. 2007). Recent studies have demonstrated that Tuina modulates glutamate concentrations and inflammatory factor levels by suppressing the expression of GluN2B and PSD‐95 in the spinal dorsal horn. This modulation leads to synaptic structural remodeling and contributes to its analgesic effects (Hongye et al. 2023). Our findings demonstrate that, compared with the CCI group, Tuina intervention significantly reduced the expression levels of GluN2B, PSD‐95, GluR1, and p‐CaMKII in the ACC. These molecular changes suggest that Tuina may modulate synaptic plasticity in the ACC by regulating the expression of the GluN2B and GluR1, thereby exerting analgesic and anxiolytic effects.

Tuina, a therapeutic modality rooted in traditional Chinese medicine, is widely recognized as a safe and well‐tolerated intervention for managing NP. Clinical evidence demonstrates that Tuina effectively alleviates both pain and associated negative emotional states in patients with knee osteoarthritis and nonspecific chronic neck pain (Xu et al. 2023; Cheng et al. 2022). However, the therapeutic efficacy of Tuina in NP comorbid with anxiety, as well as its underlying molecular mechanisms—particularly those within the central nervous system—remains to be fully elucidated. Recently, the Min Fang team demonstrated using rs‐fMRI that Tuina alleviates pain hypersensitivity in rats with NP through promotion of cerebral cortical remodeling (Z. Wu, Guo et al. 2023). This study examined the effects of Tuina on pain‐related behaviors and anxiety‐like behaviors in CCI rats. Following confirmation of Tuina's therapeutic efficacy, we subsequently targeted the ACC to investigate its underlying analgesic and anxiolytic mechanisms. The results of this study demonstrate the following: (1) Behavioral experiments (MWT, TWL, OFT, and EPM) indicate that Tuina exerts both analgesic and anxiolytic effects. (2) TEM reveals that Tuina alleviates CCI‐induced ultrastructural damage in synaptic structures. (3) Whole‐cell patch‐clamp recordings of mEPSCs show that Tuina reduces the CCI‐mediated enhancement of excitatory synaptic transmission in the ACC. (4) Mechanistically, Tuina may exert its analgesic and anxiolytic effects through modulation of the PSD‐95/GluN2B/CaMKII/GluR1 signaling pathway in the ACC.

Our study has several limitations. First, although our findings indicate that Tuina therapy can modulate excitatory synaptic transmission and structural plasticity in the ACC of CCI rats, the precise molecular mechanisms underlying these effects require further elucidation. Further investigations employing chemogenetic approaches combined with targeted pharmacological interventions would be valuable for clarifying these mechanisms. Increasing the sample size for neurobiological measures will also be essential to enable robust correlation analyses and strengthen the mechanistic interpretation of Tuina's therapeutic effects. Second, although this study focused on the impact of Tuina on anxiety‐like behaviors in NP models, it is important to note that depressive‐like behaviors often develop during the progression of chronic pain. Thus, future research should include longer observation windows and expanded behavioral assessments to more comprehensively evaluate the efficacy of Tuina in alleviating pain‐related emotional disturbances.

## 5 Conclusion

Tuina may exert its analgesic and anxiolytic effects by modulating enhanced excitatory synaptic transmission and restoring abnormal synaptic structural plasticity in the ACC of CCI rats. To the best of our knowledge, this is the first preclinical study to systematically investigate the therapeutic effects of Tuina on the comorbidity of NP and anxiety. Our findings offer robust scientific evidence supporting the application of Tuina in the management of NP accompanied by anxiety disorders.

## Author Contributions

**Rentuya Na**: formal analysis, methodology, writing – original draft. **Yue Xu**: investigation, validation. **Tianyuan Yu**: conceptualization, project administration, writing – review and editing. **Yufeng Gao**: data curation, methodology. **Yingqi Zhang**: methodology, investigation. **Jiawang Yan**: data curation, validation. **Hongzheng Zhang**: data curation, investigation.

## Conflicts of Interest

The authors declare no conflicts of interest.

## Peer Review

The peer review history for this article is available at https://publons.com/publon/10.1002/brb3.70965.

## Supporting information




**Supplementary Material**: brb370965‐sup‐0001‐SuppMat.docx

## Data Availability

All the data information is in the manuscript.
