# Supplementary material for: Tuina Alleviates Anxiety‐Like Behaviors Associated With Neuropathic Pain Through the Modulation of Synaptic Plasticity in the Anterior Cingulate Cortex
Source: Brain Behav. 2025 Oct 15;15(10):e70965. doi: 10.1002/brb3.70965 (PMC12528543; doi:10.1002/brb3.70965)
Supplement: Supplementary file 1 — Supplementary Material: brb370965‐sup‐0001‐SuppMat.docx [file BRB3-15-e70965-s001.docx]

**Key resources table**

| REAGENT or RESOURCE | SOURCE | IDENTIFIER |
| --- | --- | --- |
| Antibodies | | |
| PSD-95 | Affinity,china | AF7839 |
| GluN2B | Servicebio,china | GB115472 |
| Phospho-CaMKII alpha/delta (Thr286) Antibody | Affinity,china | AF3493 |
| GluR1 | Affinity,china | AF6306 |
| β-actin | Servicebio,china | GB15003 |
| GAPDH | Servicebio,china | GB15004 |
| Chemicals | | |
| TTX | Taizhou Kangte Biotechnology Engineering Co., Ltd.,china | Batch No.201206 |
| Bicuculline | MedChemExpress, USA | HY-N0219 |
| CGP 54626 hydrochloride | MedChemExpress, USA | HY-101378 |
